# Organic Waste Buyback as a Viable Method to Enhance Sustainable Municipal Solid Waste Management in Developing Countries

**DOI:** 10.3390/ijerph15112483

**Published:** 2018-11-07

**Authors:** Hiroshan Hettiarachchi, Jay N. Meegoda, Sohyeon Ryu

**Affiliations:** 1 United Nations University (UNU-FLORES), 01067 Dresden, Germany; 2 New Jersey Institute of Technology, Newark, NJ 07032, USA; meegoda@njit.edu; 3 Department of Political Science and International Studies, Yonsei University, Seoul 03722, Korea; sohyeon.r@gmail.com

**Keywords:** biogas, buyback programs, Cajicá municipality in Colombia, compost, developing countries, informal sector, municipal solid waste (MSW), organic waste, source separation, South Africa, sustainable development goals (SDGs)

## Abstract

Many developing countries have inadequate Municipal Solid Waste (MSW) management systems due to lack of not only the awareness, technologies, finances, but also a proper governance that is able to enforce and monitor the regulations. Not all the solutions practiced by and in developed countries fit to the developing country contexts. The local conditions and limitations must always be taken into account when proposing waste management options for developing countries. The excessively high organic waste fraction in MSW and relatively inexpensive labor markets available in developing countries are two of the strengths that have not yet been utilized fully. This manuscript is an attempt to point out the benefits we receive from the above two strengths if we establish organic waste buyback programs. This can only become successful if we find solutions to: (1) collect source-separated organic waste, and then (2) find stable markets for the products made from organic waste. Compost or biogas could be the best bet developing countries can consider as products. However, there must be some policy interventions to support buyback programs at the waste collection stage as well as at the product marketing stage. Implementation of such organic waste buyback centers that can offer some incentives can indirectly motivate residents to do source separation. This will in turn also help promote more recycling, as any waste bin that has no organics in it is much easier for anyone (e.g., waste pickers) to look for other recyclables. Developing country settings such as the Green Container composting program in Cajicá, Colombia, and buyback centers in South Africa that are presented later in the manuscript are thought to be the places where the concept can be implemented with little effort. The environment, economy, and society are considered to be the three dimensions (or pillars) of sustainability. Interestingly, the organic waste buyback centers solution has positive implications on all three aspects of sustainability. Thus, it also supports the 2030 Agenda of the United Nations (UN), by making specific contributions to the Sustainable Development Goals (SDGs) such as zero hunger (SDG 2), affordable and clean energy (SDG 7), climate action (SDG 13), clean water and sanitation (SDG 6), and sustainable cities and communities (SDG 11).

## 1. Background

In developing countries, Municipal Solid Waste (MSW) management usually does not receive the attention it needs due to many reasons. Lack of awareness, affordable/adaptable technology, finances, and proper governance are among the main reasons. Mereki et al. [1] identified lack of cooperation among stakeholders, institutional structural weaknesses, lack of legislated recycling, and ad hoc and uncoordinated approaches as key issues of improper governance. Inefficient and/or insufficient management of MSW can result in environmental as well as public health issues. Social and economic trends have implications on the characteristics, composition, and the volume of the MSW. In addition, the elements of global change—population growth, urbanization, and climate change—are making MSW management more complex than it used to be.

Developed nations have used various strategies to overcome the issues related to awareness, technology, finances, and governance in MSW management. There are many lessons that can be learned from strategies developed and used by developed countries and developing countries have also benefitted from some of those solutions. Solutions become more successful when they are tailor-made to the country perspective. However, oftentimes the socioeconomic differences make those solutions harder to prosper in developing countries. Having a “working business model” is the key to the success of any sustainable waste management project. The business model should fit the local context and focus on the “strengths” in the local environment. In this manuscript, an attempt is made to propose a conceptual solution specifically designed based on two such strengths: one related to the MSW and the other is related to the MSW workforce.

The solution that is proposed is organic waste buyback programs. A buyback program in a nutshell is a mechanism to collect something back from the users in exchange of a reward. The strength related to MSW that will be focused on is the availability of a high organic content. MSW from developing countries and regions usually has a higher percentage of biodegradable (organic) material compared to that from developed countries [2,3,4]. From the material resource point of view, organic fraction can provide nutrients to support agriculture and/or produce energy through biogas [5,6]. The strength related to the workforce that will be expected to utilize is the inexpensive labor usually available in the developing world, which can be leveraged positively towards waste sorting/recycling [7]. This can also be combined with the role played by the informal sector (mainly the waste pickers) in recycling in developing countries, which is usually not visible or measurable by the measuring indicators developed in/by developed countries. Within this context, the aim of this manuscript is to introduce waste buyback programs at a conceptual level.

Before providing details of the proposed concept, it is important to understand the characteristic features of MSW and the general status of WSM management in developing countries. A reasonable discussion on this topic is provided in the next section. This information will also help justify the reasons for why buying back should be an attractive and feasible option. Two case studies with comparable features are also presented later in the manuscript.

## 2. MSW Management in Developing Countries

**Figure 1:**
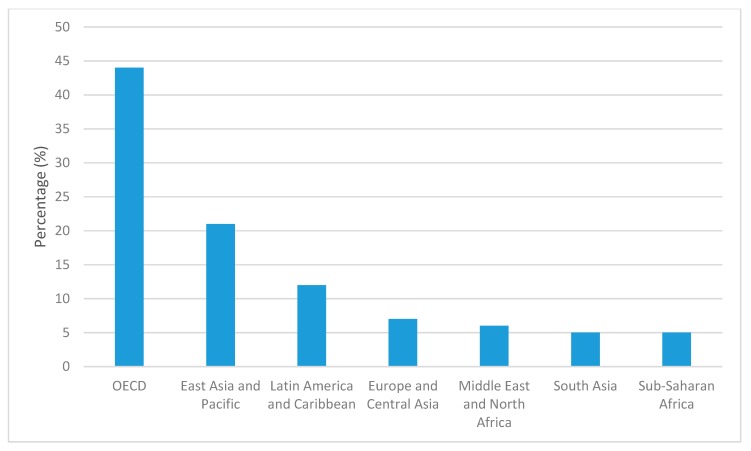
Distribution of global waste generation between different regions [2]. OECD: Organization for Economic Co-operation and Development.

Approximately 3.5 million tons of MSW is generated daily on a global scale [2]. The geographical distribution of this waste volume depends on both the developmental status and population. This can be explained with the waste generation data routinely collected and published in the World Bank reports [2]. The total MSW generated in different regions are compared in Figure 1 as a percentage of the total. The World Bank usually aggregates data for the OECD (Organization for Economic Co-operation and Development) countries in one bloc and all other countries based on their regions. The OECD bloc produces MSW at a rate of 2.2 kg/capita/day [2]. The rate is quite low in developing countries, varying from 0.45 to 1.1 kg/capita/day [2]. However, with a considerably high population, non-OECD countries still generate about 55% of the total (Figure 1). Many of the non-OECD countries are developing countries.

The regional variation of MSW composition is depicted in Table 1. It should be noted that the OECD bloc does not necessarily represent any specific region. However, for socioeconomic reasons, it is useful to compare the statistics of the OECD bloc with other regions mainly comprised of developing countries. One characteristic difference in composition is the percentage of organics and paper waste in MSW. While plastics, glass, metal, and “other” categories show a similar trend universally, organics and paper composition in MSW is different between developed and developing countries. As per Table 1, while OECD countries only report 27% organic waste fraction, all other regions shown in the table report 47–61%. According to another report by the Asian Development Bank [8], the organic fraction in MSW in South Asia might be as high as 70%. This solidifies the argument that waste from developing countries often has high organic content compared to that in developed countries [2], which in turn makes implications on MSW management decisions. For example, while the high organic content can assure a steady supply of raw materials for composting or a biogas plant, the same waste might not be the best for a waste-to-energy type of solution as it is too wet [9].

### 2.1. Collection, Recovery, and Disposal

As shown in the studies conducted by the World Bank [2] a significant portion of MSW (30–60%) is not subjected to any collection in developing countries. These figures were questioned by Wilson et al. [10] based on studies conducted by UN-Habitat covering twenty cities in six continents, which indicated 70–100% collection coverage in the upper-income countries [11]. Even the low-income cities recorded a 45–60% waste collection. Countries in the Latin America and Caribbean region (LAC) have especially made remarkable progress to achieve a coverage of 89.9% on average [12] and even reaching the universal coverage of 100% in some cities [13]. However, it should be noted that the average values in the reports do not necessarily show the socioeconomic disparities that exist in large cities in the developing countries. While the central business district and the rich neighborhoods receive 100% coverage, the poor and/or illegal settlements may not receive any coverage [10,14]. Inaccessibility to standard methods of transportation is one of the main reasons behind the lack of collection coverage in poor illegal settlements. Use of bicycles and tricycles, to bring MSW from such difficult areas to the nearest transfer stations, has become a successful solution in some Asian cities [10,15].

MSW disposal is also an issue for many developing countries. Though MSW disposal practices have improved drastically in middle-income countries since 1990s [10], open dumping and open burning are still prevalent MSW disposal methods in low-income countries [2]. Landfilling and thermal treatment have become essential components of the safe final waste treatment technologies used by the developed world. It is not financially feasible for many developing countries to adopt these technologies. Even if the capital costs were covered, lack of operating costs have led some of these projects to fail [10]. For example, sanitary landfilling practices have been developed assuming strict environmental regulations, that many developing countries lack the capacity to enforce. Another example is a challenge faced by waste-to-energy projects in developing countries due to higher moisture content in the waste and poor management of emissions. Any solution that does not fit reasonably well to an existing system has a higher chance for failure.

Since source separation is not practiced, many developing countries miss the opportunity to enhance MSW management practices through recycling and recovery [2]. Pre-disposal recycling and recovery options reduce the volume of the waste that needs further treatment in addition to the income it may generate. Although formal recycling efforts are rare to find mainly due to the lack of source separation, recycling does happen throughout the developing world thanks to the informal sector [7]. The contribution of waste pickers/scavengers in recycling, and thus to the circular economy, is huge but it is usually not appreciated, simply because there is no data on the informal sector. The presence of the informal sector and its current contribution to the local economy must be considered when new waste management projects are launched in such places. A sudden removal of their services can certainly create a negative impact on local economies. On the other hand, the informal sector presents a cost-effective labor force for any new waste management programs, if carefully designed to incorporate them.

### 2.2. Carrot and Stick Approach: Does It Work for MSW Management?

It is worth asking the question, why MSW management has taken a back seat in developing countries. The most common answers we often hear are the lack of awareness, affordable/adaptable technology, and finances. However, issues are deep-rooted and mainly related to the inability of having a functioning system within the limits of their finances, thus, with the use of technologies that they can afford, and with control measures that they can maintain. Most developing countries also have appropriate laws and regulations to manage MSW, but lack of enforcement and monitoring often prevents achieving desired results. If not enforceable, laws and regulations are not sufficient; there is a need to add other measures to motivate all stakeholders. Arguably, the right combination of governance and incentives, or more popularly (and incorrectly) known as the “carrot and stick” approach, should be the way for successful MSW management. Incentives need to be introduced in conjunction with the laws and regulations. Properly designed incentives ideally assist in encouraging the public to adhere to the desired laws and regulations at the lowest overall cost to society.

The introduction of a functional governance structure which is backed by the appropriate laws and regulations usually is the initial approach to solve any environmental problem. One of the defined branches of governance is bureaucratic governance, in which authorities and rules are used for organizing principles to achieve common social goals [7]. Bureaucratic governance of MSW management in developing countries suffers severely, especially from the limitations in enforcing policies due to weak governments [7]. The legislation on MSW management is usually weak and MSW has traditionally been the responsibility of local municipalities which brings limited revenue. Most of them are not able to collect taxes/fees for the waste management services provided by the city. Sometimes, the cities also must provide services to populations that do not exist or are not accounted for due to unplanned settlements and slums, etc. Inability to establish a financially self-sustaining service structure is an obstacle, coupled with the lack of political and legal will to address the need for adequate waste treatment. Persisting laws are also not able to address other issues such as corruption and lack of long-term political commitment. The roles, responsibilities, and the allocation of responsibilities are also often not clearly defined, creating partial overlapping of authorities [7,16].

MSW management in developing countries consumes about 20–50% of the local government budgets, and yet the service coverage is not guaranteed for the entire population [17]. This percentage seems a large proportion, but it does not tell the whole story. What they spend on MSW management (20–50%) may look bigger as a percentage but might be smaller in terms of the amount, because the total budgets are not that large. With such limitations in financing, they are not able to afford options that involve equipment, vehicles, and technologies developed in developed countries. Even if such technology is adapted with the help of international developmental/aid organizations, sustainable maintenance of such technologies is always challenging. Therefore, correct adaptation of solutions is important.

## 3. Organic Waste Buyback

The new idea we propose in this manuscript is organic waste buyback. There are different types of buyback programs that are currently in use and getting popular. The best example is perhaps the bottle return programs where customers can return plastic/glass/aluminum bottles/cans to recover the deposits that they have paid upfront. Scrap yards are still popular in many (especially developing) countries where people can sell not only scrap metal, but also glass bottles, paper, and cardboard. Some scrap yards in these countries have staff working for them who would collect these items from door to door or from waste dumps. The above existing examples cover all major waste types represented in a waste composition diagram, except one—the organics (mainly kitchen/yard waste). Ironically, the organic fraction is the largest in many developing countries and regions. Table 1 shows that, except for OECD countries, Eastern Europe, and Central Asia, in all other regions organic content represents more than 50% of the waste by weight.

While we often see and hear about buyback programs for all other types of waste, why have we not heard anything about buyback programs designed for the largest fraction of waste? That is simply because there are no such programs as of now. Now the real question is, why? We can think of many reasons such as messiness which includes odor, leachate, and gas it produces when degrading, the vectors it attracts, health hazards, and difficulty with storing. However, the economic reasons perhaps outweigh all of the above. Organic waste does not represent one pure type of waste such as paper or plastics that may be easily (1) separated and collected and (2) re-processed to make a product that has a sustainable demand. In the event we can address the above barriers, it should not be a terribly difficult task to launch an organic buyback program. The following subsections elaborate on our preliminary thinking on measures, including policy instruments, that we can consider to overcome the above issues.

### 3.1. Source Separation of Organic Waste

The lack of source separation is one of the biggest challenges faced by the waste management sectors throughout the developing world. With the introduction of legal boundaries combined with education, many countries (e.g., Germany, Netherlands) have become successful in limiting organics going into incinerators or landfills. Developing countries lack the infrastructure to apply such systemic approach, where authorities must coordinate their plans to raise awareness and enforce rules. In such countries, what can work is incentives. As the word means, buyback should mean that those who bring the organics to a collection center should be rewarded for their action.

In a recent publication on governance aspects of MSW management, Hettiarachchi et al. [7] discussed a few similar examples in the Latin America and the Caribbean region where residents receive bags of food or vouchers for bags of waste they bring to a collection center. Organics buyback centers can follow such a modality to reward customers with cash or a voucher depending on the weight or volume of the organic material they bring in. The downside of this model is that there should be a cash budget available to pay the customers upfront (before making any money from it). Perhaps this model can work better if adopted by municipalities, where they already have infrastructure and staff for waste management—this can cut down any extra costs considerably.

Taking inspiration from the bottle deposit example, perhaps we can design a policy tool to aid this process with some cashflow. While it is extremely difficult to focus on all different types of organic waste, we know a wide majority of it is food waste. What if we ask consumers to pay upfront for the future cost of what they will throw away as food waste? The moment we buy some food such as fruits, vegetables, meat, and fish we already know that a portion of what we buy will end up in the trash for various reasons. It can be the inedible parts such as the bones of the fish, peels of the banana, or the husk of a corn. Otherwise, it can be the leftovers after a meal, expired food, or even things we throw away due to a change of mind or incorrect choice. We can add a surcharge to the price of the food based on the weight of the food that should be channeled to a fund to support (at least partially) the organic buyback programs. The surcharge should be nominal and a very small percentage such as 1–2% so that it does not become a heavy burden to customers.

Since the customers have already paid a surcharge upfront, the very same fact may encourage them to reclaim part of this money by bringing the organic waste back to a buyback center. This extra amount charged, based on the weight, may also push people to think twice about the quantity they really need before deciding to buy. If we can train people to separate their organic waste, this habit also helps them to effectively manage the rest of their waste. Imagine a waste bin that has no organics in it. It should be relatively clean and dry, and you can easily identify what else is in there. This gives us the right grounds to push for more recycling. Even if it does not happen in households, or the community does not want to invent their own broader recycling program, a clean waste bin with items that can be easily separable is a luxury for waste pickers at a dump site. The other added benefit of the organic waste buyback concept is its potential for job creation. Labor is relatively inexpensive in developing countries and there are already many involved in the waste business, making a sizeable “informal sector” (usually called waste pickers) who understands recycling and its benefits. Buyback programs have the capacity to absorb this informal sector for the mutual benefit.

### 3.2. Turning Organic Waste into Usable Products

The next question is, what can we do with the collected organic waste? Although there is an array of products including compost, biogas, biochar, biocoal, etc., perhaps the ones that best suit developing countries are compost and biogas [3,18]. Both processes are relatively cost-effective, less energy-intensive, and can be accomplished with less complicated technologies [9,18]. Therefore, there is more merit in discussing those two options further.

For such projects to be successful and sustainable, the products should find a steady market. This is where most compost projects have failed in the past. A product secures a steady place in a market based on quality and the demand. Years ago, many compost projects failed due to their low quality and presence of plastics and other unwanted material in compost made of mixed waste. If the compost is made of source-separated organic waste as suggested before, the quality will not be an issue. However, the market demand for compost can still be an issue. This is where the socioeconomic factors come into the picture. When paper, steel, glass, or plastics are recycled, they are then turned into the same material to replace what they were before, i.e., recycled glass makes new glass. Food waste (organics), on the other hand, does not make any new food. What we recycle by making compost is the nutrients part of it, which will have to compete with the mineral fertilizers which might be even cheaper most of the time, due to government subsidies [8]. If compost is the option we pick as the product we want to make from organic waste, then it is essential to use other policy instruments to make sure it receives a steady market. For example, if a municipality owns a compost plant, they can decide to use its own compost in all green spaces in the city. They may also approach other government offices and schools in the city to make sure that their compost gets priority over mineral fertilizers.

On the other hand, if there are clear signs of an unstable market for compost, it is much better to think of making biogas as this is a product that can be competitive in the market. Since our focus is MSW, the biogas option has some advantages in an urban setting. The land requirement for a compost plant is much larger than that for a biogas plant. Considering the land value in an urban and/or semi-urban area, this can easily be an issue. Contrary to what some believe, establishing a compost plant in fact demands a much higher capital and much longer return period for investment when compared to a biogas plant, assuming the same amount of incoming organic waste volume [18]. However, if we are also attempting to address job creation and absorbing the informal labor sector, biogas has less potential compared to more labor-intensive composting. Depending on recruitment from the informal sector to maintain biogas facilities that demand more specialized skill sets may become problematic in developing countries.

## 4. Case Studies: Two Examples Where the Concept May Prosper

We can find many urban waste-to-compost example projects throughout the world [18] and there are also a limited number of food-for-waste type of rewards programs in some parts of the world [7]. However, to our understanding, organic waste buyback is not practiced anywhere in its entirety. Some of the existing programs have the potential to be organic waste buyback programs not only because it might be an attractive concept, but also because the buyback concept may help some of them overcome the deficiencies they have in their current systems. In the introduction, we argued that the proposed concept may be better fitting for developing countries for two reasons: first, the high amounts of organic waste in the MSW composition, and then the relatively inexpensive labor market, especially due to the presence of the informal sector. We have selected two examples, each representing one of the above two reasons, which are presented below. In each, we briefly discuss why we think the buyback concept may be a better option for them.

### 4.1. Green Containers Program in Cajicá, Colombia

Cajicá is a small city in Colombia with a rapidly growing population and located near Bogotá—the capital of the country. It is well known for its high regional gross domestic product deriving some portion from the industrial economy of Bogotá [19]. Cajicá’s organic waste management policy has been recently recognized by UN Environment as one of the five good examples of new experiences with waste management [20]. The city has pursued organic waste source separation for over a decade now and succeeded in reducing a noticeable amount of organic waste that previously used to end up in landfills.

**Figure 2:**
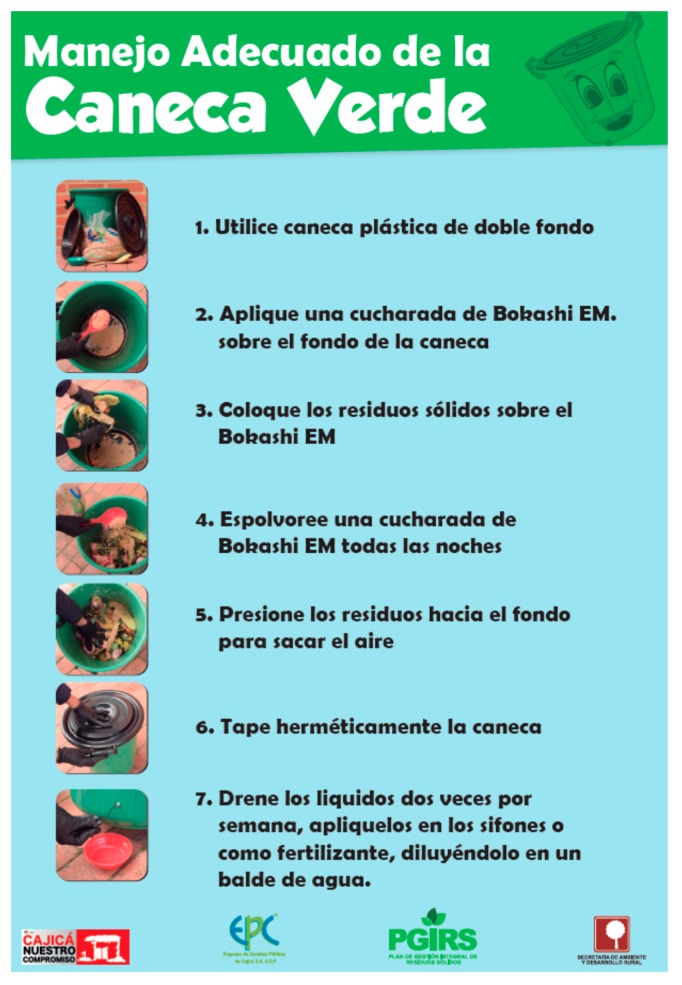
A brochure prepared by the municipality of Cajicá which explains the step-by-step procedure of the composting project to its residents (Poster courtesy of: Cajicá Municipality).

In 2005, Cajicá Municipality developed a pilot composting program [21]. This required the separation of organic waste at the source. Therefore, in parallel, they also launched an awareness program to encourage the citizens of Cajicá to sort and recycle their waste [22]. Based on the positive results observed during the pilot program, in 2008, they launched a long-term composting program nicknamed “Green Containers Program”, which has become very successful and has run smoothly ever since (see Figure 2). This program is mainly about distributing containers (green in color) for free for organic waste source separation, which is collected by the municipality once a week. The poster displayed in Figure 2 explains the steps the participants of the program should follow, with easy-to-understand pictures and simple texts. The collection is done by the same MSW management staff, which has helped the municipality to keep the (extra) cost at a minimal (information based on personal communications with Cajicá Municipality and translated by Mr. Cristian Rivera in May 2018). In addition to the containers, trained personnel from the municipality visit every household every two months to distribute a substance called “bokashi” which is a mixture of effective microorganisms. When mixed with organic waste, bokashi helps to initiate the composting process of waste while already in the green containers. In addition, bokashi is also known to decrease odors and reduces the risk of attracting pests/vectors.

The municipality has an agreement with a private vendor who turns the organic waste into compost as a business. Rather than rewarding customers—in this case, residents—with cash in return for their organic waste, they have set up a method for all customers to receive some compost each month if they desire to have them for their private use in their gardens.

Cajicá is a success story from many different angles. One of the main reasons is the high acceptance of source separation thanks to the regular training and education that targeted every household. Since the launch of the program, organic waste recovery has increased from 768 tons in 2009 to 2364 tons in 2014 [22]. The positive impact of the diversion of organic waste from landfills was clearly visible in the waste collection statistics for 2009. As a result of the Green Containers Program, there was a sudden 14% decrease in the amount disposed at landfills in 2009, just within one year since the implementation of this new program.

#### 4.1.1. High Dependence on the Municipal Government

While the program has seen its own successes, Cajicá also faces several obstacles that may hinder the self-sustainability of the collection system. We suggest that a buyback system can be a solution for them to improve self-sustainability. The first obstacle that the municipality faces is that currently the organic waste collection system is supported by the municipal government—both financially and politically. There is a substantial investment of about US$350,000 per year by the municipality to cover the cost of the program which also includes the maintenance of the educational/awareness programs (information based on personal communications with Cajicá Municipality and translated by Mr. Cristian Rivera in May 2018). This financial dependency has created a huge risk in terms of maintaining its stability, simply because any financial issues faced by the municipality can put the program in jeopardy. Also, this municipal support is vulnerable to the changes in the political arena such as changes of mayor. The mayor changes every four years, with a new staff and new goals, which means that if a new mayor does not see the program as a high priority, he or she may stop the program citing financial issues.

If they were to introduce a policy instrument where a nominal surcharge is added to their waste collection or electricity bill as this works better in the LAC region [7], that money should be able to help the program achieve financial freedom. Part of the money can be used to reward customers through food vouchers. Such a buyback system would not require external support because it operates on its own financing system and people participate by pursuing the incentive. As a result, collecting organic waste can rely less on the government’s support and the risk of financial shortage is reduced.

#### 4.1.2. Increasing Population and Waste Generation

Based on the last census conducted in 2005, the population of Cajicá was about 45,000 and projected to reach 60,000 by 2018 with 63% living in the urban areas [23]. The middle-class population is also growing according to Cajicá’s household classification statistics [24]. Following the increasing population, waste generation per capita is also rising. In 2009, one person in Cajicá produced 0.58 kg of waste per day on average. After five years, in 2014, each person generated 0.84 kg of waste a day [22]. The combined effect of increased population and per capita waste generation results in a higher waste volume within the town.

Till now, Cajicá’s compost program’s main strategy is to visit every household door-to-door to collect organic waste and educate people. However, rapid population growth and the resulting increase in waste volume implies that the current strategy might not be the best for the future. The current system requires a lot of manpower to handle organic waste from collection to processing, which will become a challenging factor to maintain when the municipality must serve a much larger population and their waste. Training its citizens to bring their source-separated organic waste to buyback centers established by the municipality may relieve the program from some of the financial obligations. A reward system such as the one explained in the previous section may encourage residents to routinely take their organic waste to the buyback centers.

### 4.2. Buyback Centers & Waste Pickers in South Africa

**Figure 3:**
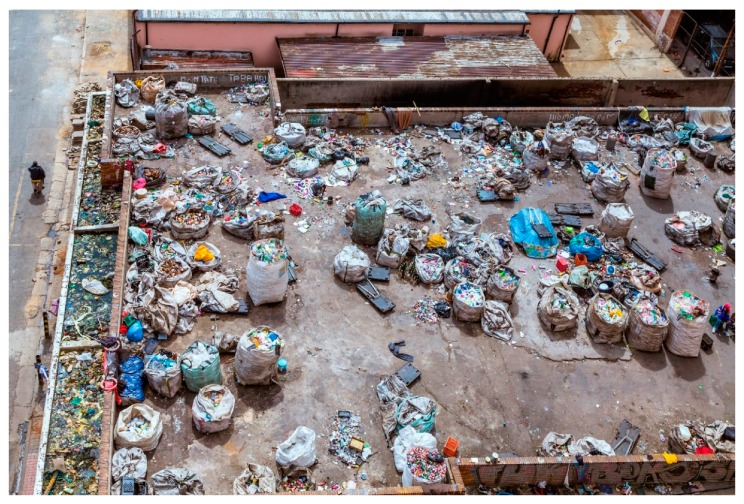
Informal sector contributions to recycling in Johannesburg, South Africa: Material collected in bags to be separated and transported to buyback centers (Photo credit: iStock photo/THEGIFT777, picture taken in March 2014).

As in many other countries, recyclable collection centers play an important role in engaging the informal sector in the recycling industry in South Africa (Figure 3). In published literature, they are popularly known as the BBCs (Buyback Centers). BBCs are privately owned commercial entities where anyone can sell their recyclable waste such as paper, cardboard, plastics, aluminum cans, glass, etc. It is not easy to judge the number of existing BBCs in South Africa due to the limitation in data availability. A study conducted by Viljoen et al. [25] on the waste BBCs in Pretoria and Bloemfontein, South Africa, reported on 10 such BBCs in Pretoria and 7 more in Bloemfontein. A total of 324 people are employed by these 17 BBCs (184 in Pretoria and 140 in Bloemfontein). They have documented how difficult it was for them to find the locations of the BBC due to lack of data. In addition to the internet, telephone directories, and referrals from the other BBCs, they also had to depend on waste pickers they found on the streets to gather more information.

These BBCs in South Africa rely heavily on waste collected by the informal sector, i.e., waste pickers. Any waste picker is welcome to bring their recyclables to the BBCs. BBCs are often located close to an industrial and/or commercial hub, making them more accessible to the informal waste collectors who do not have means of transportation for long-haul travels. The waste pickers are encouraged to bring the material clean and sort it into the different grades to ensure that the best possible price is fetched.

The recyclables collected by the BBCs are then sold to larger recycling companies. By being the “middle-man”, BBCs play a crucially important role as they form a bridge between the formal and informal sector. Though not well documented, the contribution of this informal sector (waste pickers) plays a significant role in the South African economy [26]. As per recent estimates, there are 60,000–90,000 waste pickers in South Africa [27]. The amount of money they save for the South African municipalities is close to US$47 million [28].

#### 4.2.1. BBCs’ Potential to Become Organic Waste Collection Centers

Our main reason for presenting this South African example of BBCs is that they have the right infrastructure and fitting business model to include organic waste into their collection. BBCs already function on a business model of “buying and selling waste”. They can use the same knowledge to look for the best ways to collect and then sell organic waste. This way they will continue to do the middle-man’s job by being a collection center and leave the composting or biogas production to the experts. What they will have to reconfigure is the storing of the collected material. All other recyclables do not pose odor or hygiene issues as organic waste does. That means that they need to find ways to cut down the storing time of organics at the BBC and work with buyers to come up with an optimum picking-up/delivery time. Perhaps we can learn from the previous example from Colombia and encourage the BBCs to use bins with *bokashi* in them for the temporary storing, to improve the quality control of the composting process yet also not inhibit the fermentation process during storing.

#### 4.2.2. Role of the Informal Sector

BBCs already have a team of waste pickers finding recyclables for them. They can easily train the same workforce to hunt for organic waste by adding a few minor changes to their daily routine. One such change is the locations of picking. It is not easy for anyone to pick organic waste from mixed waste in a dump or a landfill. They will probably have to look for unmixed organic waste from restaurants, supermarkets, and open markets such as farmers’ markets which are very common in developing countries. In addition to a need for more labor for picking, BBCs may need more help in-house to manage their operations. Therefore, any growth of BBC operations due to organic waste collection will certainly create new opportunities for waste pickers.

## 5. Discussion: Positive Impact of Organic Waste Buyback on Sustainability

Separation of organic waste from MSW stream at the point of generation requires buy-in from all stakeholders and implementation using only legislation is not completely effective. Therefore, policy makers need to look for out-of-box solutions to separate organic waste from MSW stream. This manuscript attempts to propose such an out-of-box solution using incentives with the aid of existing situations or modifying them. The main objective of this manuscript is not to describe organic waste buyback as a viable method using two case studies, but as an example of an out-of-box solution to inspire policy makers to implement the separation of organic waste from the MSW stream to achieve sustainable waste management.

If implemented properly, the proposed organic waste buyback concept will make a positive impact on society, the environment, and the economy which are identified as the dimensions of sustainability. Implications on the environment and economy are quite apparent; benefits to society, on the other hand, look more like a combined effect initiated by the other two. For example, the potential for job creation is an economic implication of a new organic waste buyback program, that eventually makes a positive impact on society. A change of public perception towards waste can be another such example. Training the public to do source separation is extremely difficult in developing countries. Organic waste buyback may give us an opportunity to set foot in this area in an effective way, as it may involve some sort of rewards for users.

The following sections briefly discuss the implications on the environment and economy. At the end we would also like to present a brief discussion on how the proposed idea may help us achieve the Sustainable Development Goals (SDGs).

### 5.1. Environmental Implications

Environmental and public health issues are usually ranked among the top reasons for why MSW management is important. Plastics, metals, paper, glass, etc. in MSW can become a nuisance from the management point of view; but they do not pose a serious threat in terms of public health and/or safety. Ironically, the biggest culprit in the MSW against the safety of the public as well as environmental health is the organic material in it [29]. This threat is severe in developing countries where, on one hand, the organic percentage in the MSW is high, and on the other hand, MSW management is not well organized.

We are not in a position to say that organic buyback is going to solve all MSW-related environmental/public health issues in developing countries. However, it can contribute in a significant way. Many believe that the source separation should be the starting point for any effective solution to MSW management [2,7]. The organic waste buyback concept depends completely on the source separation of organic waste, giving a good starting point for any further steps in source separation.

### 5.2. Economic Implications

**Figure 4:**
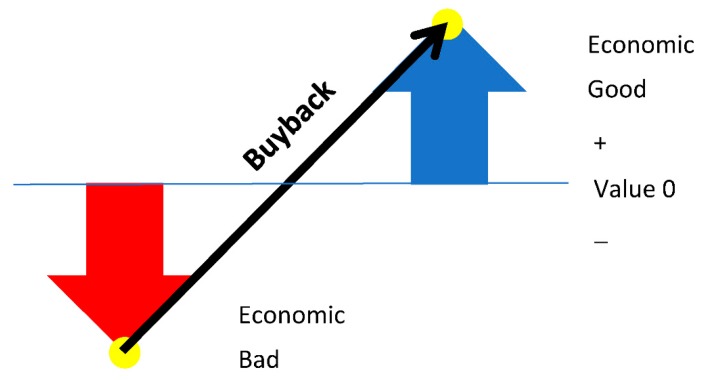
Buyback proposition can change the value of organic waste.

From the economics standpoint, organic waste buyback changes the value proposition of waste by turning it from an economic bad to an economic good (Figure 4). Economic bad, which is the opposite concept of an economic good, refers to something with a negative value to people or a negative price in the market. Economic good, on the other hand, means something that has utility and provides positive value to people. Economic bads and goods are not absolute concepts but subjective ideas that may change according to perception. Organic waste itself is an economic bad because it creates problems when not managed properly. However, for proper management with treatment, someone, often a municipality, must pay a higher price. However, a buyback system modifies people’s recognition of organic waste by returning some financial incentives. With buyback systems, people will perceive organic waste as a good with a market price and will be eager to have it for their own benefit.

### 5.3. Relevance to the Sustainable Development Goals

Looking at the 17 Sustainable Development Goals (SDGs) set by the United Nations to be achieved by 2030, we do not see any specific goal on MSW management [30]. However, many goals are somehow related to MSW, and especially organic waste can play an important role in achieving these goals. Goal 2—Zero Hunger, for example, can benefit immensely from organic waste composting. Organic waste converted to biogas is clearly an example for the solutions that we must look for in achieving Goal 7—Affordable and Clean Energy. Methane gas produced during organic waste decomposition is believed to be among the top reasons that has aggravated climate change issues. Organic waste is responsible for 16% of all anthropogenic methane emissions (Table 2) and it is the third largest producer after the petroleum and animal farming industries [31]. Any effective way to control these emissions will make a significant contribution towards achieving Goal 13—Climate Action. In addition, effective management of the organics in MSW is also important in achieving Goal 6—Clean Water and Sanitation and Goal 11—Sustainable Cities and Communities, even though the role of organics among other waste types has hitherto not been singled out.

## 6. Summary and Conclusions

This manuscript describe organic waste buyback as a viable method using two case studies, an out-of-box solution for the separation of organic waste from MSW stream to achieve sustainable waste management. MSW from developing countries usually contains a large fraction of organic material which could provide nutrients to support agriculture and/or produce energy through biogas. This is a unique feature which has not currently been used to achieve its fullest potential. The inexpensive labor market, together with the informal sector involved in recycling (waste pickers), is another untapped opportunity available in the developing world. This manuscript discussed how the above two strengths can be leveraged positively to present a viable solution to boost the sustainable MSW management activities in developing countries. The solution suggested here is to establish organic waste buyback programs combined with rewards, incentives, and policy tools that can help funnel the source-separated organic fraction of MSW to make compost or biogas. The main points discussed/revealed are briefly summarized below:

- Lack of source separation and the inability to make products that have steady market demand are the main reasons organic waste is not thought of as a sustainable resource.
- Residents in a community can be positively influenced/encouraged to do source separation if a buyback center rewards anyone who brings in pure organic waste. The reward can be in the form of cash or a voucher.
- There is room for devising appropriate policy tools to help organic waste buyback programs. Supporting buyback programs with funds raised through food sale surcharges or waste taxes collected together with other utility services, such as electricity, are some of the options.
- In addition, there should be policy instruments in place to help the new products made from the collected organic waste (especially compost) to find steady markets. Municipalities prioritizing compost over chemical fertilizer for their own needs could be one example.
- A surcharge applied to food purchases may encourage customers to do source separation, so they can reclaim part of the money they have paid upfront. It may also help us curtail food waste due to unnecessary/unplanned purchases.
- Source separation of organic waste will help us in turn to improve recycling other material; a waste bin with no organic waste is much cleaner and easier for anyone to handle, including waste pickers.
- Organic waste buyback programs create jobs and they also have the capacity to absorb the informal sector and provide them with better opportunities and working conditions.
- While organic waste buyback is not practiced in its entirety as of now, other existing buyback programs have the potential to be organic waste collection centers. A case study from South America and another one from Africa were briefly discussed.
- The proposed organic waste buyback concept can make a positive impact on society, the environment, and the economy, which are the three dimensions of sustainability.
- Sustainable management of organic waste is fundamental to achieving some of the key Sustainable Development Goals such as Goal 2—Zero Hunger, Goal 7—Affordable and Clean Energy, and Goal 13—Climate Action. In addition, it may also support us indirectly to achieve other goals such as Goal 6—Clean Water and Sanitation and Goal 11—Sustainable Cities.

## Figures and Tables

**Table 1:** Regional variation of Municipal Solid Waste (MSW) composition percentage [2].

	Organic	Paper	Plastic	Glass	Metal	Other
East Asia & Pacific	62	10	13	3	2	10
Middle East & North Africa	61	14	9	3	3	10
Africa	57	9	13	4	4	13
Latin America & Caribbean	54	16	12	4	2	12
South Asia	50	4	7	1	1	37
Eastern Europe & Central Asia	47	14	8	7	5	19
OECD	27	32	11	7	6	17

**Table 2:** Anthropogenic sources of methane (Data source: Bousquet et al. 2006).

Source	Percentage (%)
Fossil fuel production	33
Livestock farming	27
Landfills and waste	16
Biomass burning	11
Rice agriculture	9
Biofuels	4
